# Personalized Medicine in Kidney Disease

**DOI:** 10.3390/jpm13101501

**Published:** 2023-10-16

**Authors:** Guido Gembillo, Rossella Siligato, Domenico Santoro

**Affiliations:** 1 Unit of Nephrology and Dialysis, Department of Clinical and Experimental Medicine, University of Messina, 98125 Messina, Italy; 2 Department of Biomedical, Dental, Morphological and Functional Imaging Sciences, University of Messina, 98125 Messina, Italy; rossellasiligato@gmail.com; 3 Nephrology Unit, University Hospital of Ferrara, 44124 Ferrara, Italy

The Special Issue “Personalized Medicine in Kidney Disease” is focused on the importance of customized medicine in nephrology as it represents one of the main characteristics of successful therapeutic results. This edition included five articles that investigated cutting-edge subjects linked to this topic.

Personalized medicine uses the phenotypes and genotypes of individuals to tailor the best therapeutic approach for each patient at the appropriate time, to identify a person’s propensity for disease, and/or to provide timely and targeted prevention. In the context of kidney diseases, it has the potential to completely transform patient care on the basis of individual traits and needs. In particular, personalized medicine approaches in chronic kidney disease (CKD) aim to tailor treatment strategies based on underlying causes, genetic factors, the rate of disease progression, and other personalized factors.

CKD can arise through various pathological mechanisms, and it is not an infrequent result of acute renal injury (AKI). The development and progression of CKD can be related to the timing and extent of recovery of renal function after AKI. In this Special Issue, Tain et al. [1] categorized the recovery patterns after AKI within six months after hospital discharge and related them to renal function outcomes to define better strategies for personalised medicine for patients at higher risk of developing CKD.

An effective approach to limit the CKD-related injuries includes risk assessment and early identification of patients affected. The adoption of screening exams including checking blood pressure, evaluating risk factors, as well as urine and blood tests of kidney function may help to recognize people at a high risk of developing CKD and enables prompt therapy and intervention to halt illness progression. The accurate diagnosis and subtyping of CKD are also necessary for a tailored strategy; nowadays genetic testing accompanies kidney biopsy, as well as other diagnostic procedures, which can all be used to ascertain the underlying etiology of CKD. Treatment choices are likewise influenced by this knowledge, since each CKD cause may call for various management approaches.

Personalized medicine also develops treatment plans that are suited to each patient while stressing individualized lifestyle changes. Each patient’s unique demands and risk factors must be considered when selecting elements including nutrition, exercise, quitting smoking, and blood pressure control. Moreover, this method takes into account unique patient characteristics, such as kidney function, comorbidities, and medication tolerance when choosing and administering medications. In this setting, pharmacogenomic techniques may aid in particular in the discovery of genetic variations that affect drug metabolism and response.

Additionally, CKD often coexists with other conditions such as diabetes, hypertension, cardiovascular disease (CVD), and pulmonary diseases. A holistic and personalized approach addresses all these comorbidities to tailor a treatment strategy to optimize overall health outcomes. Regular follow-up visits, laboratory checks, and longitudinal monitoring enable prompt intervention and appropriate treatment plan modification. Patients should take an active role in learning about their ailment, available treatments, and associated risks and benefits, to match their therapies with their own preferences, values, and healthcare objectives in shared decision making with the nephrologists. Personalized medicine may also include the proposal of participating in clinical trials or research studies that can provide access to cutting-edge treatment approaches.

Personalized treatment for CKD should consider environmental factors that affect kidney health, including lifestyle habits like nutrition, exercise, and stress management. In this setting, a tailored approach takes into account patient preferences, socioeconomic circumstances, and cultural concerns to create customized lifestyle interventions.

In fact, prevention is essential to halt the progression of CKD and related comorbidities. Consistent with the goal of reducing the risk of AKI and progression to renal failure, Zisis et al. [2] found a potential renoprotective effect of Vardenafil and Avanafil in contrast-induced nephropathy in an animal model. Their findings may pave the way for more structured studies on renoprotective strategies to prevent contrast-induced renal damage.

One aspect that should be emphasized is the role of the kidneys in whole-body homeostasis and their subsequent connection to other organs. This side was highlighted by Gembillo et al. [3], who described the complex relationship between the kidneys and lungs and how a personalized approach should be considered in patients with multiple comorbidities. Given also the knowledge of the increasing prevalence of CKD and lung disease worldwide in the next decades, with important economic and societal challenges, the coexistence of renal and pulmonary disease demonstrates the importance of pulmonary function tests in routine clinical practice for the management of CKD patients.

Another important component of personalized treatment for CKD is represented by genomic medicine, as it is well known that genetic variables are involved in the onset and progression of many renal diseases. More and more gene mutations have been identified and linked to specific kidney illnesses thanks to the advancements in genomic sequencing technologies. Genetic testing can be used even when kidney biopsy may not provide a specific diagnosis or patients are not suitable for it, as late-referrals; it can also help in forecasting how the condition will progress, and guide therapy choices considering particular genetic mutations, for instance in the identification of potential kidney donors for transplantation and risk stratification linked to the procedure.

Studies on pharmacogenomics can provide important information on how a genetic asset impacts how each person responds to medications. Genetic variations may affect drug metabolism, its effectiveness, and side effects. Genetic information about a patient can help in drug selection and the adjustment of dosages, thus improving therapy while reducing the risk of side effects. This method is particularly important in kidney disease, since decreased renal function might affect medication clearance and clinical response.

The development of tailored therapies can be obtained using molecular profiling tools like gene expression analysis and proteomics, which can shed light on the underlying molecular mechanisms of kidney diseases. Biomarkers, as quantifiable signs that reveal information about the existence, progression, and response to therapy of diseases, are an additional helpful tool. Their role in renal disease may be to evaluate kidney function, spot early warning signals of damage, and forecast treatment outcomes.

In this context, Susilo et al. [4] adopted interleukin-6 (IL-6) as a biomarker for atherosclerotic cardiovascular disease (ASCVD) and cardiovascular mortality risk (CMR) scores in Javanese CKD patients. They also investigated the frequency of the IL-6 G174C single nucleotide polymorphism in the population. Although plasma levels of IL-6 had no direct effect on CMR, further analysis showed that they directly affected the ASCVD risk score, which in turn affects CMR.

In addition to traditional methods of obtaining real-life data, large amounts of patient data can be collected using digital health technologies such as wearables, remote monitoring and electronic health records, and finally elaborated with data analytics techniques to identify patterns, predict disease progression and improve treatment plans. Machine learning algorithms can support individualized treatment strategies by helping with risk classification and decision-making.

Personalized medicine has a lot of potential, but it has not yet been widely adopted in clinical settings. Some of the challenges include cost, the accessibility of genetic testing, data privacy, and genetic data interpretation. As we better understand the genetic and molecular mechanisms underlying kidney disease, personalized medicine techniques will become increasingly important in optimizing the treatment of patients with kidney disease and improving outcomes.

Another important factor that can contribute to the progression of CKD is hypertension. Hypertension represents a major cause of kidney disease progression. According to predictions, high blood pressure will still be the main risk factor in 2040, since it is the main cause of mortality and disability [5]. Maintaining a healthy blood pressure is influenced by vary factors, such as age, presence of comorbidities, and degree of kidney damage. A tailored approach for hypertensive kidney disease involves thus treatment strategies addressing the underlying causes, individual characteristics, and specific needs of each patient. Personalized therapy focuses on optimizing blood pressure control through lifestyle modifications and the crucial aspect of medication management, influenced by individual factors such as kidney function, electrolyte imbalances, and presence of proteinuria.

Limiting sodium intake is essential in managing hypertension and reducing fluid retention. Personalized therapy includes dietary counselling to educate patients about the importance of sodium restriction and provide practical guidance on reducing sodium in their diet. Lifestyle changes, such as adopting a healthy diet, regular exercise, weight management and smoking cessation, are integral components of personalized therapy. These modifications can help control blood pressure and reduce the risk of CVD onset.

As regards medications, clinical hypertension guidelines are broad suggestions that necessitate clinical judgment to decide how to treat best each patient, on the basis of their unique features. A treat-to-target strategy stratified by individual characteristics forms the foundation of the clinical recommendations. The causes of apparent treatment-resistant hypertension need to be evaluated in patients in whom the usual first-line medications recommended by the guidelines are ineffective so that treatment can be tailored appropriately [6,7].

Diabetic individuals have different rates of CKD progression and responses to therapy, underlining the need for individualized care [8]. A fundamental aspect in treating diabetic kidney disease (DKD) is represented by proper glycemic control to reduce the extent of kidney damage. This involves individualized diabetes management, including medication regimens (insulin or oral hypoglycemic agents), dietary adjustments, and regular monitoring of blood glucose levels.

Different comorbidities can aggravate the kidney involvement in the course of DKD. Hypertension is common in DKD and contributes to further kidney damage. Blood pressure targets and antihypertensive medications are similar to those in hypertensive kidney disease (as mentioned earlier).

Adequate proteinuria management also represents an essential element for proper disease control. Medications such as angiotensin-converting enzyme inhibitors (ACEi) or angiotensin receptor blockers (ARB) are commonly prescribed to reduce proteinuria and slow the progression of kidney damage. The choice of medication and dosing may be personalized based on kidney function, blood pressure control, and individual tolerability. An important asset in treating DKD is the adoption of sodium-glucose co-transporter-2 inhibitor (SGLT2i). The proximal convoluted kidney tubules express SGLT2 channels that oversee around 90% of the reabsorption of filtered glucose. SGLT2i blocks this activity, resulting in glucosuria and improved glycemic management [9]. In addition to this anti diabetic effect, SGLT2i also causes natriuresis, which improves blood pressure management. Moreover, through tubule-glomerular feedback, natriuresis enhances sodium transport to the juxta-glomerular apparatus reducing the glomerular hyperfiltration seen in DKD [10].

Additionally, DKD worsens dyslipidemia and increases the risk of atherosclerotic CVD. Personalized therapy may involve lipid-lowering medications to control cholesterol levels to reduce the risk of CVD. The absolute amount of cardiovascular risk and the initial lipid profile heavily influence the decision to perform lipid-modifying therapy. Most patients who need to lower their levels of low-density lipoprotein cholesterol (LDL-C) are treated with statins first, with the addition of ezetimibe and pro-protein convertase subtilisin/kexin type 9 (PCSK-9) inhibitors to help them attain their LDL-C goals. Based on phenotypic and genetic characteristics, some side effects of statins are somewhat predictable. If triglyceride levels continue to be elevated, fibrates or omega-3 fatty acids can be administered. The RNA-targeted treatments currently under research offer the potential for selective liver targeting for particular lipoproteins like lipoprotein(a), and the long-term lowering of LDL-C with occasional delivery of a small-interfering RNA, which may help to address the issue of adherence to medication [11].

Similar to hypertensive kidney disease, lifestyle modifications play a crucial role in managing DKD. These include following a balanced diet, practicing regular physical activity, weight management, smoking cessation, and stress reduction. Personalized therapy also includes the regular monitoring of kidney function, blood pressure, blood glucose levels, and other relevant parameters. This helps to assess treatment effectiveness, identify any complications, and adjust the treatment plan as needed.

For DKD patients, personalized therapy necessitates an individualized strategy that considers each patient’s special traits, preferences, comorbidities, and treatment response [12]. Collaborating with a healthcare team, including nephrologists, endocrinologists, dieticians, and other specialists, can help to develop and implement a tailored treatment plan to optimize outcomes and slow the progression of kidney disease and avoid therapeutic inertia [13].

Nutrition therapy plays a crucial role in treating kidney disease and maintaining renal function meeting the specific needs of each patient.

An important aspect of appropriate nutrition therapy regards sodium and fluid management. In particular, limiting sodium intake with a correct fluid assumption results in better control of blood pressure and congestion, thus reducing the kidneys’ and heart’s workload. The extent of sodium and fluid restriction must be tailored to the individual’s specific kidney function and the overall health of the patient.

The amount and the sources of protein intake should also be adjusted individually depending on the stage of kidney disease and the presence of proteinuria. A low-protein diet in CKD patients appears to slow the natural progression to end-stage renal disease (ESRD) but it is still not appropriate for all patients if the nutrition intervention is not personalized to ensure treatment adherence [14].

Controlling phosphorus and potassium is another crucial aspect of adequate nutrition for CKD patients. High phosphorus levels can contribute to bone and cardiovascular complications [15], while elevated potassium titers can lead to cardiac arrhythmias and other CKD-related complications [16]. Nutrition therapy considers food phosphorus and potassium content and specifies which processed foods or certain fruits and vegetables need to be restricted. Phosphorus and potassium binders are the second line of treatment, when the nutrition approach has failed to control these electrolyte imbalances.

Additionally, dietary modifications may be required to control acid–base levels depending on the patient’s unique situation. This may entail reducing consumption of foods that promote the synthesis of abnormal amounts of hydrogen ions, as this is a major factor in endogenous net acid production acid formation. On the other hand, privileging the consumption of alkaline foods such as fruits and vegetables or the oral intake of alkaline salts [17] may represent an alternative for persistent metabolic acidosis.

Individuals with CKD may also have specific nutrient deficiencies due to dietary restrictions or impaired kidney function. Supplementation of certain vitamins and minerals, such as vitamin D, iron, zinc, copper and B-complex vitamins, may be necessary to address these deficiencies [18,19,20]. However, the need for supplementation should be evaluated on an individual basis with blood testing and the assessment of overall nutritional status. From this perspective, creating individualized meal plans that consider food preferences, cultural background, and socio-economic factors can enhance the adherence to the prescribed dietary recommendations. Working with a registered dietitian specialized in kidney disease may help the nephrologist to meet the person’s nutritional needs while considering the individual circumstances.

Another important topic for CKD treatment is Personalized renal replacement therapy (RRT). RRT refers to tailoring the treatment of ESRD to meet the specific needs and characteristics of each patient. In special populations such as in older adults, personalized RRT considers age-related changes, comorbidities, performance status and the goals of care. The treatment plan is adjusted to optimize outcomes while considering the individual’s overall quality of life. In some cases, conservative management or supportive care measures may be considered as fair alternatives to aggressive RRT in older patients.

Hemodialysis (HD) represents the most common treatment modality of RRT. Personalizing HD involves optimizing dialysis parameters, such as dialysis frequency, duration, filters, fluid removal and blood flow rates, based on the individual’s clinical condition, residual kidney function, and overall health status. Modifying the HD prescription can help to improve the effectiveness of treatment and minimize complications. Incremental HD may be the preferred approach for ESRD patients who do not need an urgent start and preserve residual kidney function; in these cases, the dialysis dose is increased by degrees to allow a gradual transition that reflects the progressive nature of CKD and reduce the patient’s dialysis burden.

Peritoneal dialysis (PD) is another form of RRT that relieves patients with ESRD of the need for frequent hospital dialysis sessions and can enhance quality of life. PD is, by definition, tailored to the patient’s lifestyle, residual kidney function, and the high, average, or low transport rate of each patient’s peritoneum. A PD prescription may be personalized by choosing different dialysis solution types, dwell times, and exchange volumes, which can be adjusted to optimize treatment outcomes and patient comfort.

Moreover, personalized RRT can include the option of home-based dialysis therapies, including both HD and PD. Home-based dialysis offers more flexibility and convenience to patients as they have more control over their treatment plan and require less frequent hospital visits. The decision to perform home-based therapy may be shared based on the patient’s preferences, life habits and the presence of care givers where needed.

Kidney transplantation is considered the optimal treatment for ESRD because it offers the best long-term outcomes and quality of life. The careful assessment of potential kidney donors, taking into account factors such as blood group compatibility, human leukocyte antigen (HLA) match and the presence of sensitization or pre-existing antibodies is an example of a personalized approach. Adapting the transplant process and the immunosuppressant therapies to the specific needs of the patient can improve the chances of a successful transplant and long-term graft survival.

Personalized RRT aims to maximize the benefits of treatment while minimizing the risks and burdens for each patient. The decision-making process should involve a multidisciplinary team of healthcare professionals, including nephrologists, transplant surgeons, nurses, and dietitians, who can assess the patient’s specific needs, preferences, and goals, and collaborate to develop a unique treatment plan.

In glomerulonephritis, personalized techniques entail personalizing therapy based on an individual’s particular disease subtype, underlying causes, genetic variables, immune system traits, and patients’ requirements, which may influence therapeutic options. Precision medicine begins with an accurate diagnosis and subtyping of the glomerular diseases, with a combination of clinical assessment, laboratory tests, imaging studies, and kidney biopsy. Determining the precise subtype guides appropriate treatment strategies. Given the genetic background of some forms of glomerulonephritis, personalized medicine may involve genetic testing to identify specific gene mutations or variants associated with the disease, that may also help in predicting disease progression and assessing familial risk. In certain cases, targeted therapies or genetic counselling may be considered based on the identified genetic abnormalities.

Another aspect to investigate is represented by the immune profiling, through serological testing, measurement of autoantibodies, complement system evaluation, and other immunological assessments. As glomerular diseases are often expressions of immune system dysregulation, such as immune complex deposition or autoantibody production, a patient’s detailed profile is useful in directing targeted immunosuppressive therapies and monitoring treatment response.

Moreover, patient-oriented therapy includes the choice of different strategies based also on comorbidities, severity of disease, preferences, including pregnancy desire [21,22]. Treatment options include immunosuppressive drugs (such as corticosteroids, cyclophosphamide, mycophenolate mofetil or rituximab) in autoimmune diseases, as well as targeted biological agents, plasma exchange or supportive therapies.

It is critical to closely monitor disease activity and response to treatment through regular clinical assessments, laboratory tests and imaging studies. In this way, disease flares or progression can be detected early and the treatment plan can be adjusted as needed. Biomarkers such as proteinuria, complement levels or genetic markers can be used to assess disease activity and predict response to treatment. In this Special Issue, Aiello et al. described the prognostic and predictive role of interstitial macrophages in kidney biopsies from patients with IgA nephropathy [23]. The authors discovered that interstitial macrophage infiltration in kidney biopsies from IgAN patients was associated with microvascular rarefaction. Interstitial macrophage infiltration was found to be an independent risk factor for progressive deterioration of renal function. The combination of corticosteroids and RASi improved outcomes and displayed promising prognostic utility for risk stratification in this population. This scientific contribution may help to better tailor individual therapy and prognosis in IgAN.

The aim of personalized medicine should be to identify a patient’s predisposition for a disease and to carry out timely and targeted prevention, as well as determine the optimal therapeutic approach for each patient affected at the right time. In this context, the Special Issue “Personalized Medicine in Kidney Disease” introduces intriguing new viewpoints and highlights the importance of tailored strategies for each individual, given that treatments may vary largely according to specific kidney diseases and individual characteristics. Applying precision medicine is fundamental to develop therapeutic plans that optimize outcomes and minimize the risk of complications.
